# *Solanum lycopersicum* heme-binding protein 2 as a potent antimicrobial weapon against plant pathogens

**DOI:** 10.1038/s41598-023-47236-z

**Published:** 2023-11-20

**Authors:** Atefeh Farvardin, Eugenio Llorens, Luisa Liu-Xu, Lorena Sánchez-Giménez, Aloysius Wong, Elena G. Biosca, José M. Pedra, Eva Falomir, Gemma Camañes, Loredana Scalschi, Begonya Vicedo

**Affiliations:** 1 Biochemistry and Biotechnology Group, Department of Biology, Biochemistry and Natural Sciences, Universitat Jaume I, 12071, Castellón de la Plana, Spain; 2 College of Science, Mathematics and Technology, Wenzhou-Kean University, Wenzhou, 325060 Zhejiang China; 3 Department of Microbiology and Ecology, Universitat de Valencia, E-46100, Valencia, Spain; 4 Central Service of Scientific Instrumentation, Universitat Jaume I, 12071, Castellón de la Plana, Spain; 5 Department of Inorganic and Organic Chemistry, Universitat Jaume I, 12071, Castellón de la Plana, Spain

**Keywords:** Microbiology, Antimicrobials, Applied microbiology

## Abstract

The rise in antibiotic-resistant bacteria caused by the excessive use of antibiotics has led to the urgent exploration of alternative antimicrobial solutions. Among these alternatives, antimicrobial proteins, and peptides (Apps) have garnered attention due to their wide-ranging antimicrobial effects. This study focuses on evaluating the antimicrobial properties of *Solanum lycopersicum* heme-binding protein 2 (SlHBP2), an apoplastic protein extracted from tomato plants treated with 1-Methyl tryptophan (1-MT), against *Pseudomonas syringae* pv. *tomato* DC3000 (*Pst*). Computational studies indicate that SlHBP2 is annotated as a SOUL heme-binding family protein. Remarkably, recombinant SlHBP2 demonstrated significant efficacy in inhibiting the growth of *Pst* within a concentration range of 3–25 μg/mL. Moreover, SlHBP2 exhibited potent antimicrobial effects against other microorganisms, including *Xanthomonas vesicatoria* (*Xv*), *Clavibacter michiganensis* subsp*. michiganensis* (*Cmm*), and *Botrytis cinerea*. To understand the mechanism of action employed by SlHBP2 against *Pst*, various techniques such as microscopy and fluorescence assays were employed. The results revealed that SlHBP2 disrupts the bacterial cell wall and causes leakage of intracellular contents. To summarize, the findings suggest that SlHBP2 has significant antimicrobial properties, making it a potential antimicrobial agent against a wide range of pathogens. Although further studies are warranted to explore the full potential of SlHBP2 and its suitability in various applications.

## Introduction

According to the 2020 report by the World Health Organization (WHO), antibiotic resistance poses a significant threat to global health, food security, and development. In response to this challenge, various antibiotic alternatives have been developed, each with their own advantages and limitations. One promising alternative is the use of antimicrobial proteins and peptides (APPs), which are unique molecules with a wide range of applications. These APPs hold great potential as next-generation antibiotics to combat bacterial resistance. They are often integral components of the innate defense system in organisms, with different molecular weights. APPs can be derived from diverse sources, including bacteria, fungi, algae, plants, insects, and mammals. Alternatively, they can be synthesized as analogs of naturally occurring APPs^1^.

The recent development of strong high-throughput genomic tools has shown the presence of hundreds of thousands APP-like genes in various plant genomes^2^. APPs can be expressed constitutively in specific plant organs or induced by various stresses, including biotic and abiotic stresses^3,4^. Over 3000 experimentally confirmed APPs have been identified to date, with 70% of them showing antibacterial activity^5,6^. The defensive antimicrobial proteins produced by plants are classified into two groups based on their size. One of them is the group of large antimicrobial proteins (more than 100 amino acid residues) and includes the chitinases and glucanases that are induced in plant tissues by fungal attack. The second group is formed by smaller proteins (less than 100 amino acid residues) and are termed as antimicrobial peptides (AMPs)^7^. The antimicrobial activities and mechanisms of action of AMPs are diverse^1^.

In response to microbial infection, several APPs are secreted by the host cells in the apoplastic space. The apoplast serves as the primary site for the recognition of pathogenic microbes in many plant-pathogen interactions. It is within this compartment that secreted proteins and other metabolites from both the host and the pathogen come into contact, playing a crucial role in determining the plant's resistance or susceptibility to disease^8,9^. The pathogen-related proteins (PR-proteins) are the predominant proteins in the apoplast that play a crucial role in defense mechanisms^3^. Recently, several studies have reported the roles of apoplastic proteases in plant resistance against bacteria, fungi, and oomycetes^10–12^.

The SOUL protein family, also known as heme-binding protein HBP/SOUL, encompasses a group of evolutionarily conserved proteins that have putative heme-binding properties. These proteins are found in animals, plants, and bacterial species. Investigations into SOUL/HBP proteins in Arabidopsis unveiled their localization within organelles and their organelle-specific activities^13^. Moreover, it was observed that they are implicated in phytochrome-mediated red/far-red light responsess^14^ and the heme oxygenase-mediated antioxidant pathway^15^. However, to date, no heme-binding protein HBP/SOUL has been reported to be secreted into the apoplast, which is an important environment for leaf pathogens. Only a few of these proteins have been linked to plant defense mechanisms^16^.

In our previous study^17^, we demonstrated that the apoplast derived from tomato plants treated with the resistance inducer 1-methyltryptophan (1-MT), whether infected or not, exhibited inhibitory effects on the growth of *Pst* compared to control plants, with or without infection.

Subsequent proteomic analysis of the apoplast obtained from 1-MT-treated tomato plants, conducted by^18^, revealed the presence of plant defense proteins in this apoplastic environment, including an apoplastic protein belonging to the heme-binding protein HBP/SOUL family, termed heme-binding protein 2 (SlHBP2). Based on these findings, the aim of our current study was to investigate and characterize the antibacterial properties of SlHBP2. Our focus was to assess the effectiveness of SlHBP2 against *Pst*, providing a comprehensive evaluation of this newly discovered bactericidal compound.

## Results

### Computational assessment of the SlHBP2 heme-binding region

**Figure 1:**
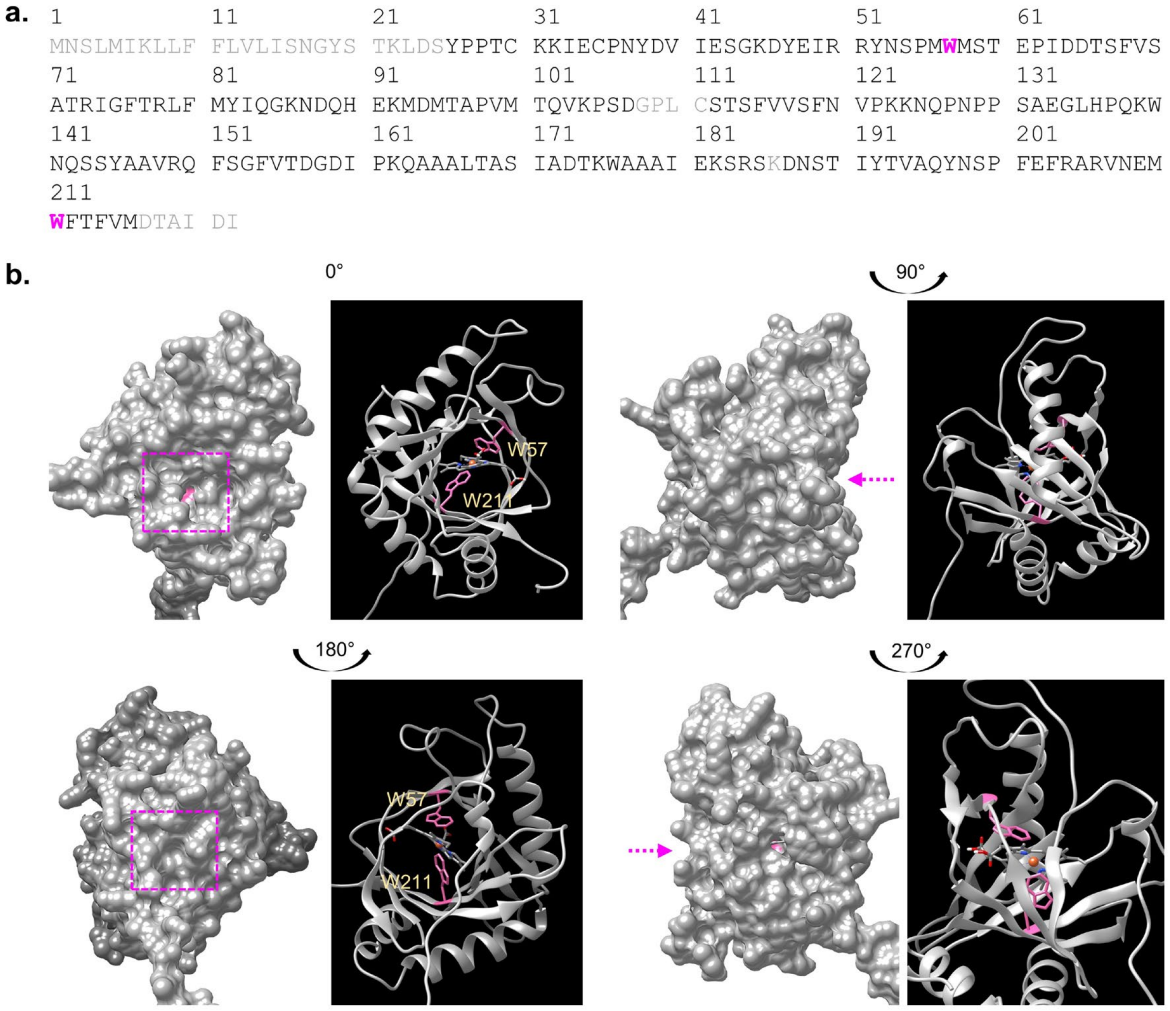
Sequence and structural analysis of SIHBP2. (**a**) Full-length amino acid sequence of SIHBP2 (UniProt: A0A3Q7HE03). The key tryptophan residues W57 and W211 identified as crucial for interactions with the heme moiety are represented in pink. Regions with grey colored amino acids have low to very low model confidence (pLDDT < 70) whereas regions with black colored amino acids have high to very high model confidence (pLDDT > 70), based on the protein structure prediction generated by AlphaFold (ref). pLDDT, which is scaled from 0 to 100, is an estimate of the confidence of each amino acid corresponding to the model’s predicted score on the lDDT-Cα metric^19^. Except for the N-terminal 20-amino acid long signal region (marked in grey), the model, which includes the key tryptophan residues at the heme binding pocket, has mostly high to very high confidence score, and is deemed suitable for characterization of ligand or cofactor binding sites. (**b**) Computational assessment of the heme-binding region of SlHBP2. The SIHBP2 model generated by AlphaFold obtained from https://alphafold.ebi.ac.uk/entry/A0A3Q7HE03^19^ and was used for structural assessment and docking simulation studies. The heme-binding region of SlHBP2 is shown within the pink box as surface model (left) and as ribbon model (right). Docking simulation with AutoDock Vina indicates that the heme-binding region occupies a distinct pocket in the model (pink arrow and pink box), and importantly, this cavity can spatially accommodate heme. The tryptophan residues W57 and W211, are identified as likely amino acids for interaction with the heme moiety. Docking simulations were conducted using AutoDock Vina (version 1.1.2)^20^ and structural visualization and image preparation were performed using UCSF Chimera^22^.

The full-length protein sequence of *Solanum lycopersicum* HBP2 (SlHBP2) is shown in Fig. 1a. This sequence was used to query the NCBI database, and the retrieved hits show high degree of conservation across species from bacteria to animals and plants. Based on sequence homology, this protein is annotated as SOUL heme-binding family protein and the closest homolog in *Arabidopsis thaliana* is AtHBP1 (NP_173153.1/At1g17100.1) which has a 58.71% sequence identity to and covering 89% of the queried sequence. However, no crystal structures of plant HBP proteins were available. Thus, to structurally analyze the heme-binding region of the SlHBP2 protein, the 3D model generated by AlphaFold was obtained from https://alphafold.ebi.ac.uk/entry/A0A3Q7HE03^19^ (Fig. 1b). Except for the N-terminal 20-amino acid long signal region, the amino acids in the entire heme binding pocket of this model, has mostly high to very high confidence score, which is deemed suitable for characterization of ligand or cofactor binding sites^19^.

Based on the 3D model of SlHBP2, a distinct cavity resembling that of other HBPs was observed as indicated by the pink squares in Fig. 1b, and this is the only pocket spatially large enough to accommodate the heme moiety. To determine the feasibility of heme-binding to this region of SlHBP2, molecular docking with AutoDock Vina^20^ was performed. Docking simulations with SlHBP2 as the receptor and heme as the ligand revealed that SlHBP2 can accommodate heme at the predicted pocket with W57 and W211 appearing as key amino acids for interaction with the heme (Fig. 1b). It is noted that the available crystal structures of HBP are apoproteins with no heme attached, thus our docking simulations provide some clues on how heme may dock to HBPs at the molecular level. In particular, we identified the two tryptophan residues, W57 and W211, as likely amino acids for the coordination of the heme group in the pocket, conceivably participating in tryptophan to heme electron transfer much like those observed in ferrous myoglobins^21^.

### SlHBP2 binds heme in vitro

Structural studies of SlHBP2 revealed that the two tryptophan residues, W57 and W211, are essential for heme-binding. Thus, to investigate whether SlHBP2 can bind heme directly we expressed the protein in *E. coli* Bl21 DE3 cells and checked up its heme-binding properties. For this purpose, the purified recombinant SlHBP2 protein was incubated with hemin-agarose. Last wash and eluted fractions were analyzed by Sodium dodecyl-sulfate polyacrylamide gel electrophoresis (SDS-PAGE). As it can be seen in Fig. S1, the result of SDS-PAGE, showed that SlHBP2 bound to hemin-agarose and was detected in the eluate fraction while no band was visible in the negative control. In conclusion the data strongly show that SlHBP2 binds heme in vitro.

### SlHBP2 exhibits strong antibacterial activity against *Pst *in vitro

**Figure 2:**
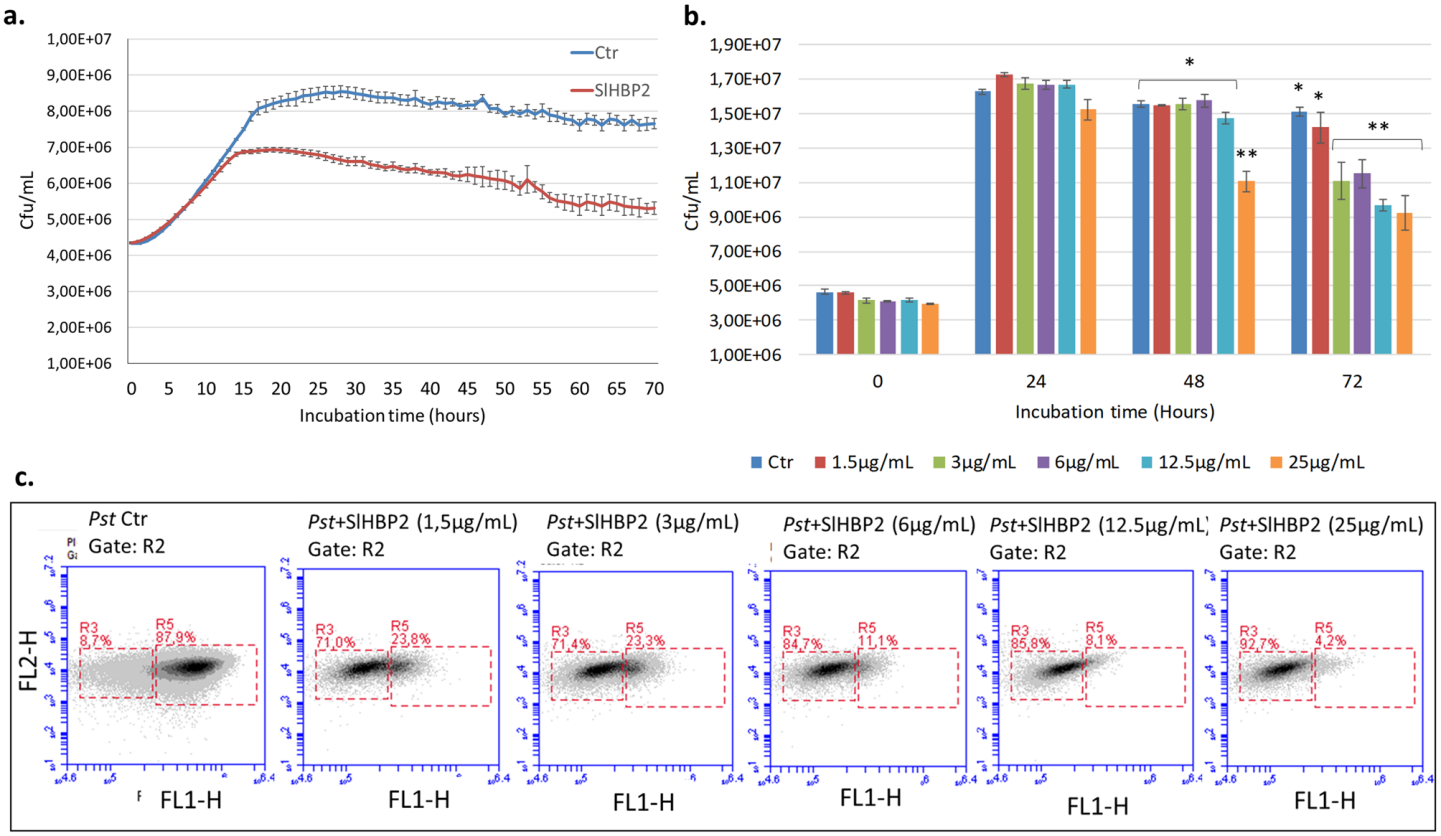
Effect of the SlHBP2 protein on the growth of *Pst*. (**a**) Antimicrobial effect of SlHBP2 at 75 µg/mL. (**b**) MIC and (**c**) MBC results. All the assays were performed in M9 minimal medium in a Multiskan plate reader under constant agitation, at 28 °C, taking measurements at 600 nm every 10 min for 72 h. The asterisks indicate statistically significant differences between groups (*P* < 0.05; least-significant difference test). After SlHBP2 exposure cell samples were stained with Live/dead BacLight™ and measured by flow cytometry. R3 represents the area with dead bacteria, and R5 represents the area with live bacteria in Ctr, 1,5 µg/mL, 3 µg/mL, 6 µg/mL, 12,5 µg/mL, and 25 µg/mL, respectively. The results of one experiment are shown. Four independent experiments were carried out with similar results.

We next decided to check whether SlHBP2 has antibacterial activity in vitro against the Gram-negative bacterial pathogen *Pst*. A clear inhibitory action on the growth of the bacterial population was observed over time at a concentration of 75 µg/ml (Fig. 2a) since bacterial populations stopped growing after 14 hpi in the presence of the protein. These experiments were repeated three times obtaining similar results.

Once the inhibitory action of the protein was demonstrated, two different approaches were applied to characterize the SlHBP2 antimicrobial properties, Minimum Inhibitory Concentration (MIC) and Minimum Bactericidal Concentration (MBC). For MIC determination, bacterial growth was measured for 72 h in M9 minimal medium amended with different concentrations of the protein (25 µg/mL, 12,5 µg/mL, 3 µg/mL and 1,5 µg/mL) or with phosphate buffered saline (PBS). It was observed that SlHBP2 inhibited *Pst* growth at 25 µg/mL since the cells showed normal growth in the first 24 h of the assay followed by a rapid decline of the culture starting from this time point. At concentrations lower than 25 µg/mL, the cells showed normal growth until 48 h and afterwards started to decline except for the 1,5 µg/mL concentration where the cells showed normal growth along the whole assay. These results show that SlHBP2 inhibits the growth of *Pst* in a concentration dependent manner and that, the MIC value is 3 µg/mL (Fig. 2b).

However, although the growth of the bacteria was reduced in the samples incubated in the presence of the protein, these results cannot rule out the fact that the bacterial cells could have entered a viable but nonculturable state. To check whether the effect of the treatment was bacteriostatic or bactericidal, the samples were stained with the LIVE/DEAD BacLight kit and further analyzed through flow cytometry as seen in Fig. 2c. It can be observed that after 72 h of incubation, in the control samples there was a quantity of living cells of around 90% while in the samples incubated with the protein, the percentage was reversed, being proportional to protein concentration. Concentrations of 3 and 6 µg/mL already killed more than 60% of the bacteria while at 12,5 and 25 µg/mL a percentage of dead cells of 83% and 92% was observed respectively. Taking into account that MBC was defined as the lowest concentration of protein at which more than 90% of the cells were dead, the 25 µg/mL concentration could be considered as MBC.

### Heme group site-directed mutagenesis does not affect SlHBP2 antibacterial properties

**Figure 3:**
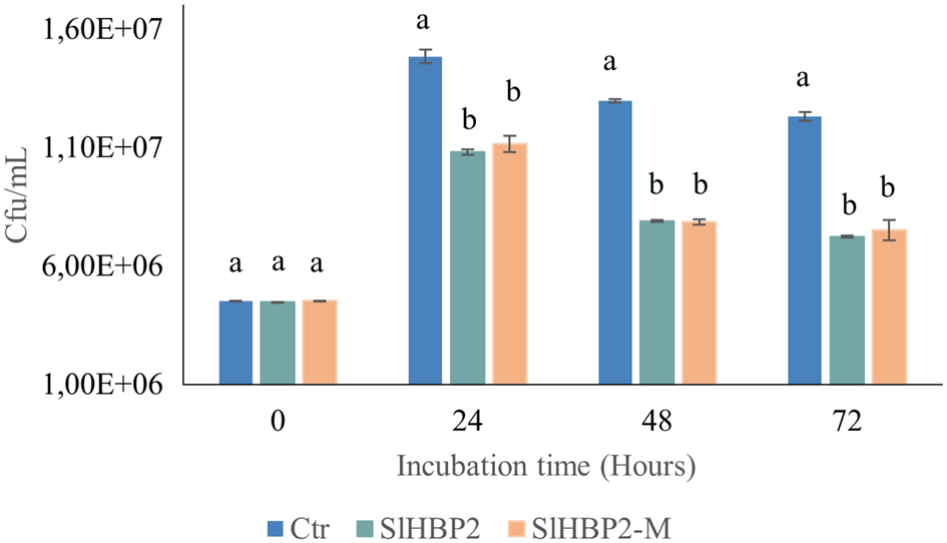
Comparative antibacterial properties of SlHBP2 and SlHBP2-M (heme group mutant) against *Pst.* A concentration of 75 µg/mL of SlHBP2 and SlHBP2-M was applied in M9 minimal medium inoculated with *Pst*, and the culture was kept at 28 °C under continuous agitation for 72 h. Graph shows the means with the standard errors. Different letters represent statistically significant differences (*P* < 0.05; least-significant difference test). Four independent experiments were carried out with similar results.

To investigate the potential role of the heme group in the antibacterial properties of SlHBP2, mutagenesis of the tryptophan residues, W57L and W211L, that appeared as key amino acids for the interactions with the heme moiety, were generated and the obtained protein was named SlHBP2-M (Fig. 3). Study of the antibacterial activity in M9 minimal medium revealed that SlHBP2-M exhibited similar antibacterial activity to SlHBP2.

### SlHBP2 accumulates in *Pst* cytoplasm

**Figure 4:**
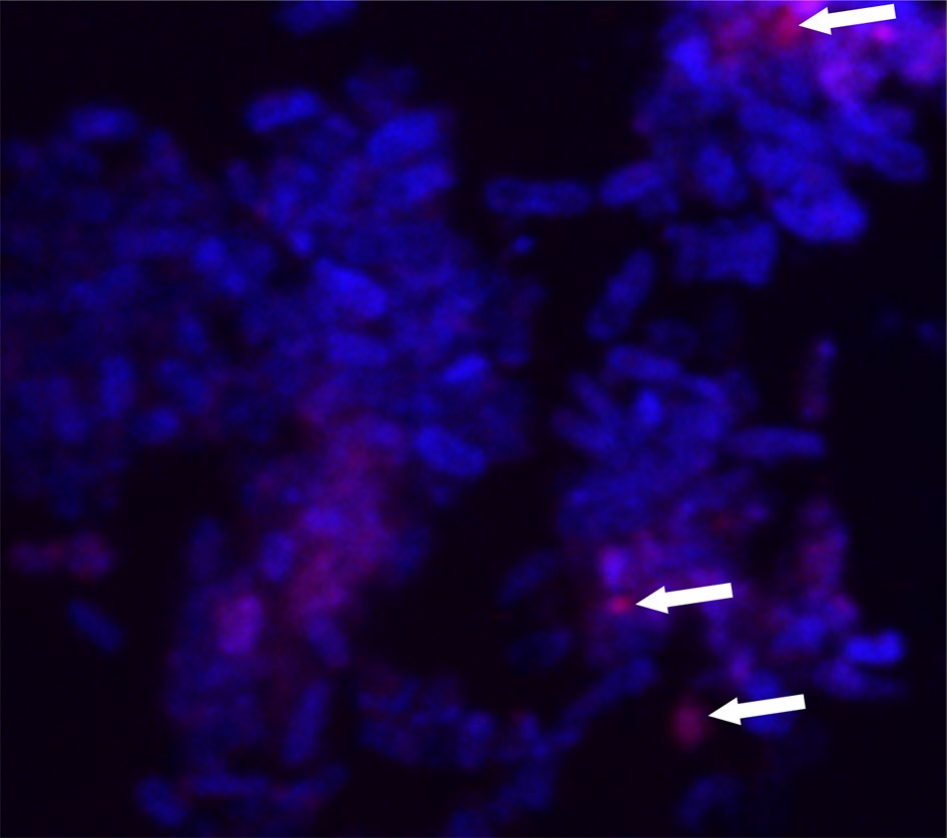
Localization of SlHBP2. Fluorescence confocal microscopy images of bacteria treated with SlHBP2 and stained with Alexa Fluor 647-conjugated anti-His secondary antibody and with Hoeschst 33,342. Pink fluorescence indicates the localization of the SlHBP2 in the cells. Blue fluorescence shows cytoplasmic DNA stained with Hoeschst 33,342.

To test if SlHBP2 interacts only with the bacterial membrane or enters the cytosol, confocal microscopy was performed on bacterial cells grown for 16 h in the presence SlHBP2 and further stained with Alexa Fluor 647-conjugated anti-His secondary antibody and with Hoeschst 33,342 solution marking the SlHBP2 in pink and the bacteria DNA with blue color, respectively (Fig. 4). It was observed that Alexa Fluor 647-SlHBP2 penetrated through *Pst* membranes and accumulated in the bacterial cytoplasm. Intracellular localization was further investigated by examining co-localization of Alexa Fluor 647-SlHBP2 with Hoechst 33,342, which stains DNA. The fluorescence of Alexa Fluor 647-SlHBP2 and Hoechst 33,342 labels did not overlap, supporting that SlHBP2 localization is cytoplasmic.

### SlHBP2 induces morphological changes in the cellular wall of the bacterium

**Figure 5:**
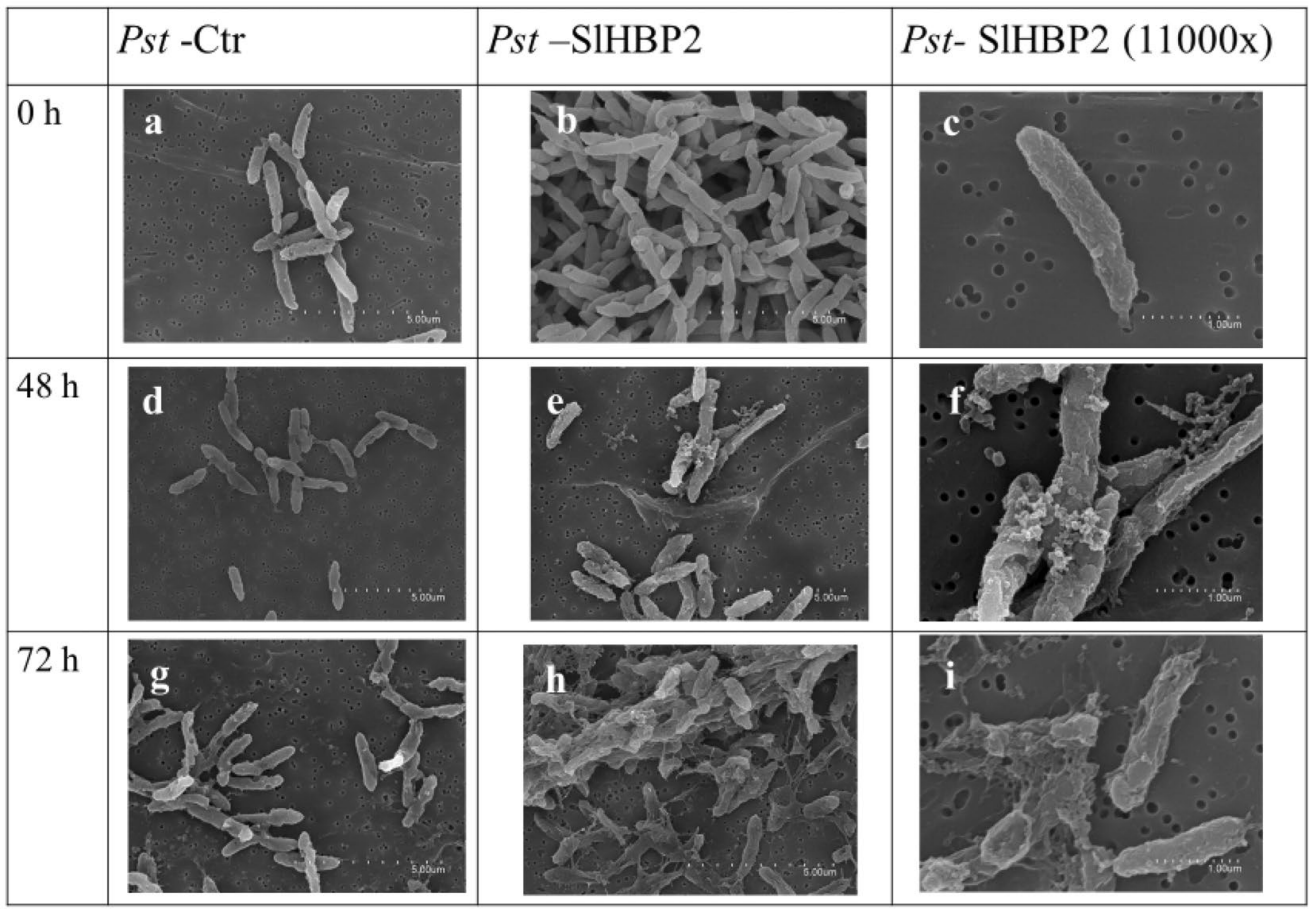
SEM micrographs of untreated *Pst* (*Pst-*Ctr) and of SLHBP2 treated *Pst* (*Pst*-SlHBP2) after 0, 48 and 72 h of incubation in M9 minimal medium. The untreated *Pst* cells were long, intact, and evenly shaped at all studied time points (**a–d–g**). SlHBP2 treated *Pst* cells show loss of cell wall integrity starting from 48 h while 72 h treatment with the protein caused complete lysis of *Pst* cells (**b–e–h).** Higher magnification images of samples (**e–f–i**).

The morphological changes of the cells treated with SlHBP2 were observed with scanning electron microscopy (SEM) at 0, 48 and 72 h of incubation (Fig. 5). The results showed clear differences in the cell wall morphology between the control or the SlHBP2 treated cells. The control group kept their fundamental morphology and had an intact cell wall, and the bacterial surface was smooth at all studied time points (Fig. 5a,d,g). On the contrary, after 48 h of treatment with SlHBP2 the *Pst* cells showed vesicle on the surface indicating the loss of cell wall integrity (Fig. 5e,f). Moreover, the accumulation of cell debris as a result of cell lysis was observable around some of the cells at the same time point while, at 72 h past SlHBP2 treatment, a complete lysis of the bacterial cells was observed (Fig. 5h,i).

### SlHBP2 increased the biofilm formation

Biofilm is produced by bacteria as a protective mechanism against external agents. This study aimed to investigate the influence of SlHBP2 on the prevalence and behavior of *Pst* by assessing its biofilm-forming ability (Fig. S2). Biofilm accumulation was quantified using crystal violet assays, which measure the absorbance at 550 nm. The results showed that, in the presence of SlHBP2 the biofilm formation was increased 1.5-fold when compared to the control, suggesting a substantial enhancement in biofilm production. These results indicate that the presence of SlHBP2 induces a stress response in *Pst*, leading to increased biofilm formation.

### Raman spectroscopy characterization of *Pst* cells exposed to SlHBP2 treatment reveals damage in the cell membrane and cell wall

**Figure 6:**
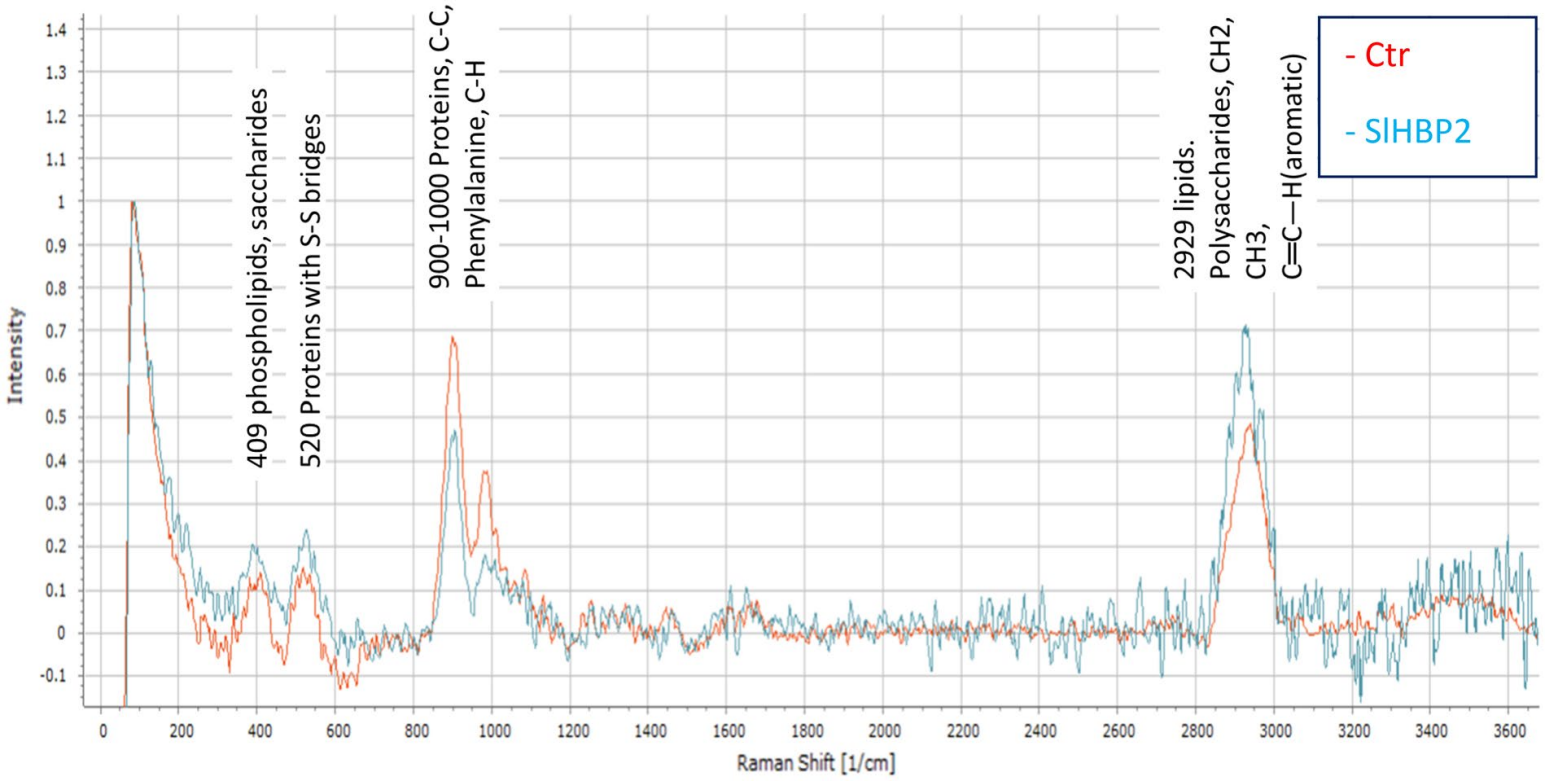
Raman spectra of control *Pst* (red line) and SlHBP2 treated *Pst* (blue line). The assay was repeated three independent times and the spectra are the average of those assays. The y-axis is in arbitrary intensity units and the x-axis is in wavenumbers (cm^−1^).

To gain additional information on SlHBP2 mode of action, biochemical properties of *Pst* cells grown in the presence or absence of the protein were determined using Raman spectroscopy. Figure 6 shows the averaged Raman spectra of *Pst* cells undergoing control (red) or treatment with SlHBP2 (blue). The width Raman band in the Raman spectra for *Pst* cells undergoing control treatment 900–1000 cm^−1^ corresponds to proteins (C–C;phenylalanine;C-H). The strong band at 2929 cm^−1^ could be associated to lipids and polysaccharides (CH_2_, CH_3_ and C=C–H(aromatic)). The peaks at 409 cm^−1^ can be assigned either to phospholipids or to saccharides while the peak at 520 cm^−1^ belongs to proteins containing S–S bridges^23^. To check the effect of the treatment with SlHBP2 on *Pst* cells components, we next determined the Raman spectra for these cells. *Pst* cells upon exposure to the treatment showed a decrease in the magnitude of Raman intensity at 900–1000 cm^−1^ corresponding to proteins. On the contrary, the magnitude of Raman intensity at 2929 cm^−1^ associated with lipids and polysaccharides increased following treatment with SlHBP2. An increase was also observed in Raman intensity for the bands at 409 cm^−1^ and 520 cm^−1^, respectively. In addition, new bands appeared in the wavenumber interval 500–700 cm^−1^ corresponding to COC glycosidic ring ref (540 cm^−1^), Phenylalanine (620 cm^−1^) Tyrosine (640 cm^−1^) and to DNA/RNA (Guanine (665 cm^−1^), Adenine (720 cm^−1^), Cytosine, Uracil (785 cm^−1^)) indicating the release of intracellular components that points to cell damage. Moreover, in the range 2650–3200 cm^−1^ of the spectrum, bands with higher Raman intensity were observed for *Pst* treated cells. These bands are typical for CH_2_ (2870–2890 cm^−1^), CH_3_ and CH_2_ (2935 cm^−1^) and C=C–H (aromatic) groups, which could indicate a release of lipids and polysaccharides probably coming from the membrane and cell wall. Therefore, the results of the RAMAN indicated that not only a disintegration of the membrane occurred, but also that the integrity of the cell wall has been lost and, consequently, molecules contained in the cytoplasm have been released.

### SlHBP2 treatment can alter the expression of bacterial virulence genes in vitro

**Figure 7:**
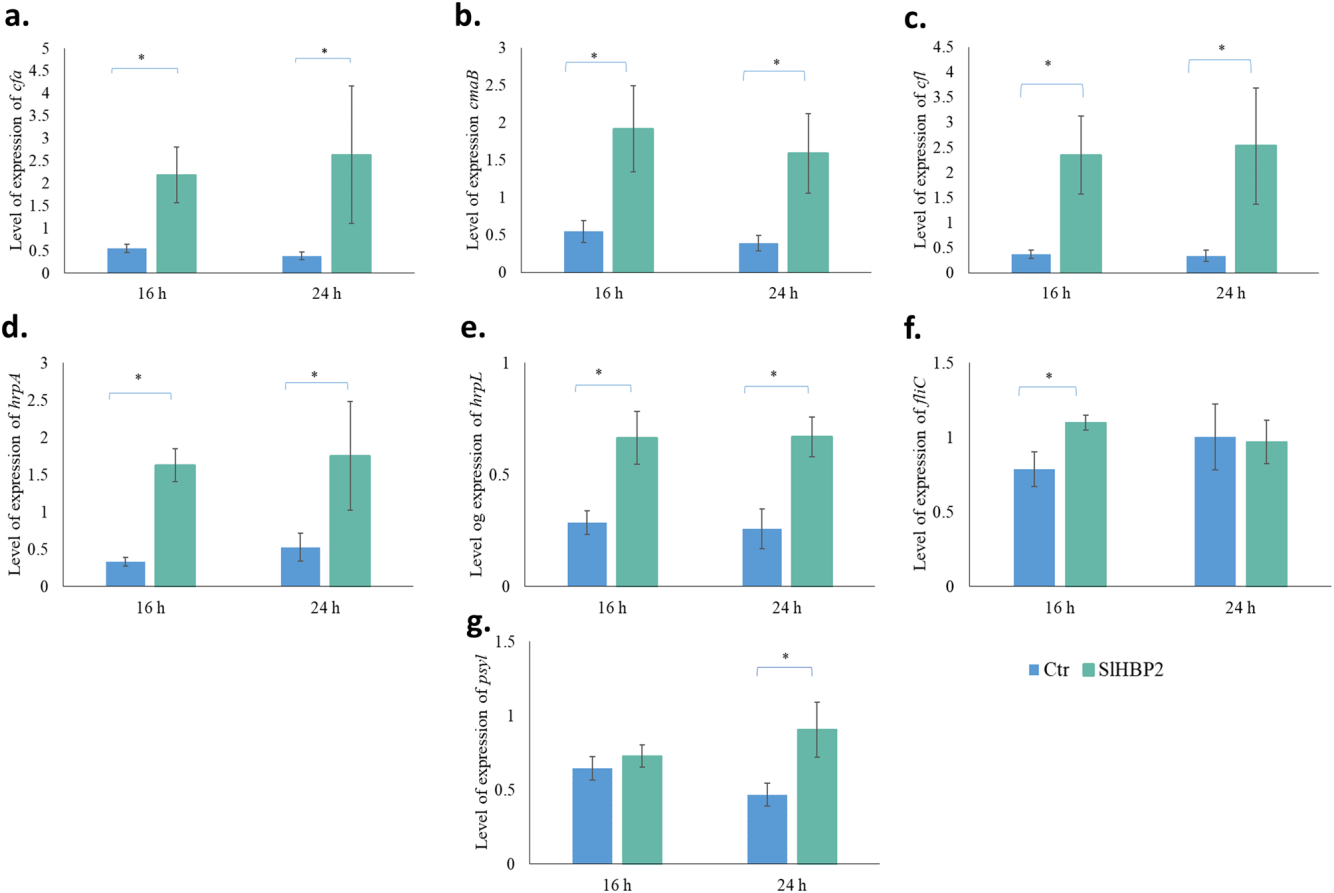
Differential gene expression was assessed by RT-qPCR after *Pst* were cultured in the presence of 50 µg/mL of SlHBP2. (**a, b, c**) show the synthesis of coronatine (*cfa, cmaB, cfl*) genes, (**d, e**) represent T3SS marker genes (*hrpA, hrpL*), and (**f, g**) represent *fliC* and *psyI* genes respectively. The samples were collected 16 and 24 h Bars represent relative expression changes of target genes in bacteria grown in the presence of the antimicrobial agent , with significance indicated by asterisks (*P < 0.05, Student's t-test).

We also performed in vitro assays to determine SlHBP2 effect on bacterial gene expression. For this purpose, the bacteria were cultivated in M9 minimal medium for 16 h and 24 h, both in the absence or presence of SlHBP2. The selected genes and their functions are described in Material and Methods section. The results showed a similar behavior of the studied genes at both time points except for *fliC* and *psyI*. Presence of SlHBP2 resulted in a fourfold increase in the relative expression of the genes involved in the synthesis of coronatine (*cfa*, *cmaB*, *cfl*) (Fig. 7a,b,c) and in the Type III Secretion System (T3SS) marker genes (*hrpA*, *hrpL*) (Fig. 7d,e) indicating an activation of these genes in response to the stress caused by the treatment. On the other hand, although the expression of *fliC* gene has also been upregulated in the presence of SlHBP2 at 16 h, no differences were observed between treated and control samples at 24 h, (Fig. 7f). Furthermore, when the relative expression of the *psyI* gene was analyzed, no significant differences were observed between bacteria incubated with or without the protein at 16 h, while at 24 h an increase in *psyI* expression was observed in the presence of the protein (Fig. 7g).

### A broad—spectrum antimicrobial activity of SlHBP2

**Figure 8:**
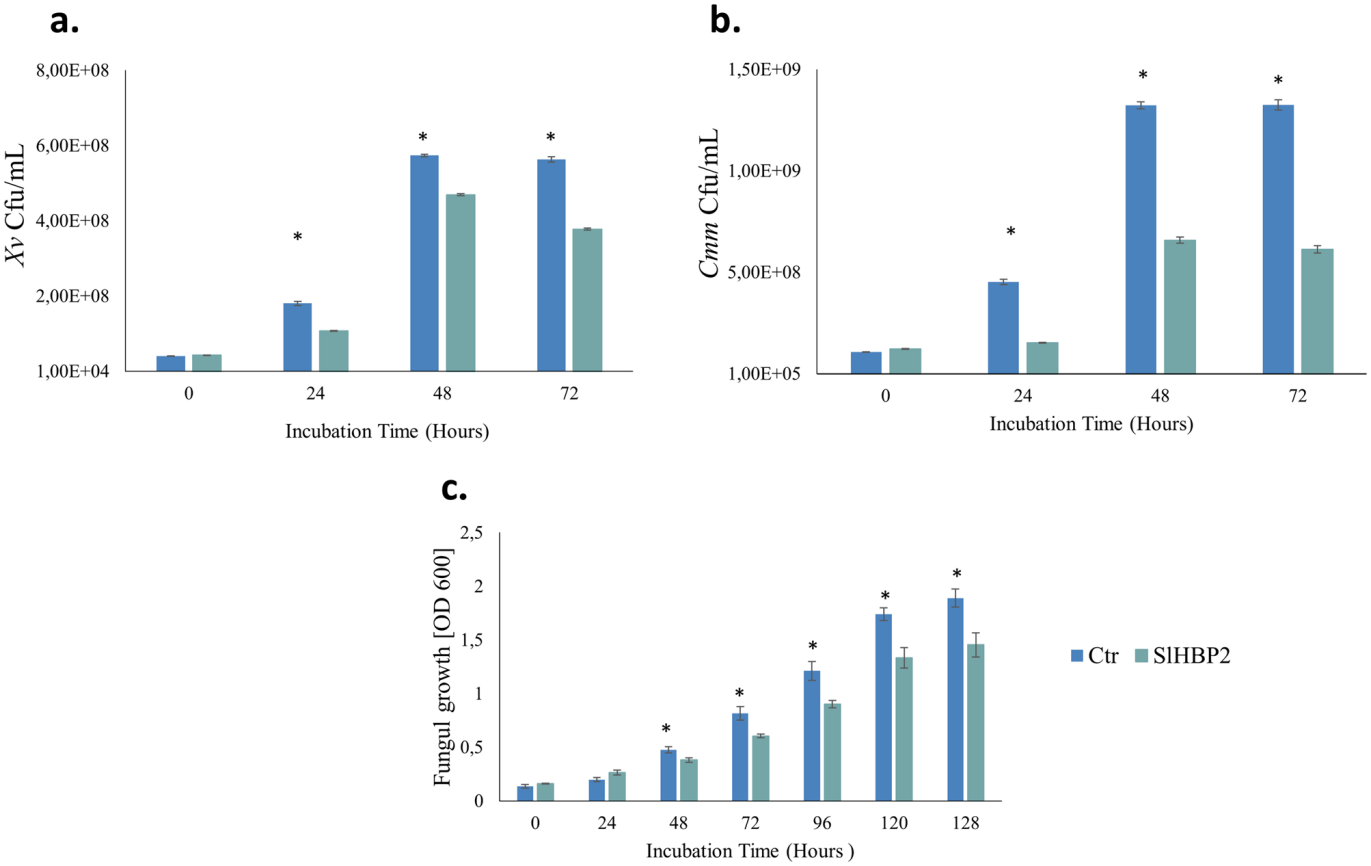
The effects of SlHBP2 on different plant pathogens. (**a**) *Xv*, (**b**) *Cmm*, and (**c**) *B. cinerea.* Growth was assessed through optical density measurements (OD) at a wavelength of 620 nm and 492 nm for bacteria and fungi respectively. Normality tests were performed, and significant differences (*P* ≤ 0.05) were determined using one-way ANOVA for parametric data and the Kruskal–Wallis test for non-parametric data. The asterisks indicate significant differences between groups at each time point.

In order to examine the impact of SlHBP2 on different microorganisms, we specifically chose the gram-negative bacterium *Xv*, the gram-positive bacterium *Cmm*, and the necrotrophic fungus *B. cinerea,* known for their significance in causing plant diseases. Our experimental results represented notable variations in the growth patterns of *Xv, Cmm,* and *B. cinerea* when subjected to SlHBP2, as illustrated in Fig. 8. Notably, *Xv* displayed a 1.5-fold reduction in growth, when exposed to SlHBP2 for 72 h (Fig. 8a). Similarly, *Cmm* exhibited a 2.1-fold decrease in Cfu/mL compared to the controls after 72 h in the presence of SlHBP2 (Fig. 8b). Additionally, the growth of *B. cinerea* exhibited a significant decline following 48 h of SlHBP2 treatment that was maintained during the whole assay, indicating a noticeable contrast to the control (Fig. 8c).

## Discussion

In the quest for sustainable plant pathogen control, researchers have turned to naturally derived compounds as antibiotic alternatives^24,25^. Antimicrobial Proteins and Peptides (APPs) have emerged as promising candidates, with diverse mechanisms of action^26,27^ ,and demonstrated effectiveness against different type of plant pathogens^28–30^, this research represents the antimicrobial capacity of novel APPs with strong antimicrobial activity which have never been tested before.

In this study we have cloned and purified SlHBP2 which was detected in the proteomic study in the apoplast extracted from tomato plants treated with the inductor 1-MT, independently of infection to test its mode of action as a plant defense protein.

Based on sequence homology, SlHBP2 was identified as a SOUL heme-binding family protein with two tryptophan amino acids (W57 and W211) that probably coordinate the heme group in the SlHBP2 pocket. Although proteins adopt the same fold, a comparison of our results with binding data acquired for the homologous protein SOUL revealed that proteins use various ligand binding sites. In contrast to SlHBP2, in which two tryptophan amino acids interact with the iron and hold stabilizing heme moiety, SOUL uses Histidine as an iron ligand^31^.

Purified recombinant SlHBP2 (expressed without the signal peptide) bound to hemin-agarose in vitro. This is a strong indication that SlHBP2 also acts as heme-binding protein in vivo*.* As previously described, heme plays an active role in primary plant metabolic pathways as well as in stress signaling. Hemes are synthesized in plastids but are also required for vital cellular activities in numerous organelles. For example, hemes play a critical role in several biological processes such as respiration, protein targeting, transcription and translation regulation, ion channel regulation and signaling, microRNA processing, and protein degradation^32–34^. Moreover, Wu et al.^16^ have observed that two heme-binding protein-like proteins, A0A1U8EZE6 and A0A1U8EZN6 were induced by *B. tabaci* in a resistant variety of pepper but not in a sensitive one suggesting their role in mitigating the oxidative stress produced by the insect when penetrating the plant.

We next tested the potential antimicrobial activity of SlHBP2. We observed that the antibacterial activity was significantly enhanced with increased in concentration from 3 to 75 µg/mL. Furthermore, our results revealed that the key amino acids in the heme environment did not alter the bactericidal properties of the protein, suggesting that the heme and concomitantly also iron binding, may still be intact and that these residues may instead participate in other molecular functions such as electron transfer, such as that observed in ferrous myoglobins^21^. Consistently, several previous reports have assigned specific roles for iron binding during infection^35,36^. For instance, lipocalin-2 decreased the growth of *E. coli* H9049 by reducing the availability of iron during infection of mice^37^. Also, Schaible et al.^38^ associated the antibacterial effect of lactoferrin to its iron-binding property as observed during *Mycobacterium tuberculosis* infection. Furthermore, the antibacterial ability of lactoferrin against *Pseudomonas* spp. including *P. fluorescens* and *P. syringae* was observed^39^.

Furthermore, several experiments have been conducted to determine the protein's mode of action. One of these was the determination of cell viability by flow cytometry which is widely used to assess membrane disorders that are associated with cell death caused by antimicrobial agents^40,41^. This method is based on propidium iodide (PI) uptake which freely penetrates cell membranes of dead or dying cells but is excluded from viable cells. Our results showed that *Pst* treated with SlHBP2 were able to take up more PI dye than the control. Many studies have confirmed that when PI binds to DNA, it produces a lot of fluorescence, which shows that the integrity of the inner membrane has been damaged^42^. In this work, a great membrane instability was observed at higher doses of the protein, boosting bactericidal efficiency. These results were consistent with previous studies on AMPs^43,44^.

Confocal microscopy is widely used to visualize APPs localization within a bacteria cell^45^. We observed the signal within the cytoplasm of *Pst* cells, which points that SlHBP2 crossed the bacteria cell membrane. Similarly, Li et al.^46^ reported that CF-14, a tomato plant protein, had antibacterial activity against *E.coli,* a gram-negative bacterium, by localization in cytoplasm.

Besides, SEM was used to prove the damaging effect of SlHBP2 on the bacterial cell wall. The SEM image showed that, in response to SlHBP2, the bacterial cell wall of *Pst* displayed wrinkles, tiny vesicles in the short time and even rupture at more long time, compared to the control group. In addition, treatment with SlHBP2 caused the leakage of the cell content, proving significant damage to the *Pst* cell wall, membrane permeabilizing ability and cleavage activity. These findings were comparable to many other studies that determined the bacterial cell wall and membrane damage caused by AMP treatment using SEM^47,48^. These experimental results were consistent with those of growth curves and PI uptake in flow cytometry, confirming that SlHBP2 presence causes cell damage.

Moreover, the presence of vesicles on the bacterial surface upon the treatment can be associated with the response of the bacteria to a hostile environment^49^. It seems that the bacteria, in the presence of the protein, responds to the stress caused by it, which could explain the increased expression of the defense genes such as those responsible of the synthesis of the T3SS or the genes involved in the synthesis of coronatine. This response has already been observed in previous in vitro studies, in the presence of hexanoic acid, a molecule with antibacterial effects^50^, which reinforces the role of virulence systems as defensive systems in response to antibacterial molecules. The fact that the expression of the flagellum synthesis gene is initially activated but at long terms is similar to the control, and that the synthesis of signal molecules is activated over a longer period of time, together with the results obtained for biofilm formation, suggest that the bacterium tries to synthesize biofilm to protect itself, as a defensive response against the stress caused by the presence of the protein. Several authors correlate the presence of vesicles on the surface of the bacteria together with the secretion of signal molecules for biofilm formation^51^ with defense against different stresses such as the presence of antibiotics and peptides^52,53^.

To better understand the antimicrobial mechanisms of action of SlHBP2 against *Pst*, Raman Confocal Microscopy studies were carried out. Results of chemical characterization of *Pst* cells exposed to SlHBP2 treatment by Raman spectroscopy confirm the cell damage produced by the presence of the protein. To understand to which chemical structures each observed peak corresponds, we have based ourselves on the previous description of other authors like Sahoo et al.^54^ and Czamara et al.^55^. The observation of differences in the two curves of the Raman spectrum provided valuable insights into structural changes in bacteria when grown in the presence of SlHBP2 compared to control bacteria. The analysis revealed distinct regions in the spectrum where untreated cells exhibit higher peak intensities, particularly between 850 and 1050, which could indicate a higher cellular density associated with protein abundance^54^. On the other hand, the results revealed that treated bacteria showed an increased presence of phospholipids, lipids, and polysaccharides. Given that the bacteria are Gram-negative, this suggested a disruption of the cell wall and membrane integrity. Moreover, the presence of proteins with disulfide bonds in the treated samples could be a response to the stress induced by the protein, as the bacteria attempt to defend themselves. It is known that bacteria induce the formation of disulfide bonds to facilitate the release of periplasmic proteins, such as oxidoreductase disulfide bond protein A and B (DsbA and DsbB). These proteins catalyze the formation of disulfide bonds in various protein substrates located in the periplasm of Gram-negative bacteria^56^. Several studies by different authors have demonstrated the relationship between the presence of these proteins and their impact on the production of disulfide bonds in other proteins present in the periplasm, as well as on the bacterial response to oxidative stress^57^. Additionally, the higher presence of proteins in the untreated bacteria, specifically those different from cysteine-rich proteins, could explain the degradation of cellular components in the treated bacteria that hinder their detection.

## Conclusion

In conclusion, our studies highlight the significant antimicrobial properties of SlHBP2, a heme-binding apoplastic protein isolated from tomato plants, specifically against *Pst* as well as other microorganisms like *Xv*, *Cmm*, and *B. cinerea* which points to a possible use against a wide range of pathogens. SlHBP2 demonstrated remarkable efficacy in inhibiting the growth of *Pst* by disrupting bacterial cell walls and causing intracellular content leakage. Understanding the in vitro mechanism of action against the pathogen is just the first step; it is crucial to explore how this protein performs when applied to plants and how the plant responds to the treatment. Consequently, the authors of this study are currently designing future experiments to investigate the role of this protein in plant defense, which will provide valuable insights into the protein's in vivo mechanisms of action. Future works that focus on experimentally resolving the structure of SIHBP2 especially in the presence heme in its unligated and ligated forms and in the presence of different ligands, would reveal the molecular mechanism that may contribute to potential modification and application of SIHBP2 as a potent antimicrobial agent.

## Material and methods

### Plasmid construction

Primers used in this study are listed in Supplementary Table S1. All constructs were sequence-verified to confirm their integrity before further manipulation. For protein expression and purification, the sequence encoding the mature SlHBP2 **(**GeneBank accession number XM_004244269.4) without the signal peptide was amplified by PCR using Phusion® High-Fidelity DNA Polymerase (New England Biolabs) in a 50 μL reaction volume containing 10 µL of GC buffer, 1 µL of 10 mM dNTP, 2.5 μL of 10 µM of each primer and 1 µL of DNA template, 0.5 µL of Phusion® High-Fidelity DNA Polymerase, 1.5 µL DMSO and 31 µL of nuclease free water. The following conditions were applied: initial denaturation of PCR product was at 98 °C for 30 s, followed by 35 cycles; denaturation at 98 °C for 10 s, annealing at 60 °C for 20 s, elongation at 72 °C for 30 s. The program was followed by final elongation at 72 °C for 2 min. The PCR products were separated on a 1.0% agarose gel, purified with a Macherey–Nagel™ NucleoSpin™ Gel and PCR Clean-up kit, ligated with pJET plasmid (Thermo Scientific), and transformed into competent *E. coli* DH5α cells by heat shock methods^58^.The recombinant plasmid pJet–SlHBP2 was further digested with *BamHI* and *XhoI* restriction enzymes, and the SlHBP2 fragment was ligated into the expression vector, pET-14b (Novagen) linearized with *BamHI* and *XhoI* The pET-14b-SlHBP2 plasmid was transformed into competent *E. coli* BL21 (DE3) cells for expression of the desired protein.

The expression vector of the mutated SlHBP2 was constructed in a similar way. Briefly, mutagenesis of the tryptophan residues at the N′ and C′ end (W57L and W211L) was generated by site-directed PCR mutagenesis. The resulting fragments of 125 bp (containing W57L) and of 512 bp (containing W211L), were joined together by using the Gibson Assembly Master Mix kit (New England Biolabs) and further cloned into pET-14b.

### Protein purification

Expression and purification of the recombinant proteins were performed using Ni–NTA resin (Thermo Scientific) according to the manufacturer instructions. In brief, 50 mL of LB medium (supplemented with 100 μg/mL ampicillin) was inoculated with 0,5 mL of pre-cultured *E. coli* BL21 strain containing the desired plasmid and the cultures were incubated at 37 °C with shaking until they reached OD600nm = 0.6. The culture was cooled to 16 °C, IPTG (isopropyl-β-D-thiogalactoside) was added to a final concentration of 0.2 mM, and the cultures were incubated with shaking overnight. The cells were harvested by centrifugation (5000 rpm, 4 °C, 15 min) and then resuspended on ice in equilibration buffer (PBS with 10 mM imidazole, pH = 7.4). The cells were then disrupted by sonication. The bacterial cells sonication lysates were centrifuged (12,000 × g, 45 min at 4 °C,), and the supernatants were loaded onto the Ni–NTA columns previously equilibrated with equilibration buffer. The columns were washed with wash buffer (PBS with 25 mM imidazole; pH 7.4) and eluted with elution buffer (PBS with 250 mM imidazole; pH 7.4). The proteins were further cleaned by using a Zeba Spin desalting column (Thermo Scientific).

### Heme-binding assay

Heme-binding assay was performed as follows: purified SlHBP2 was mixed with 100 µl hemin agarose pre-equilibrated with equilibration buffer (50 mM TRIS–HCl, pH = 8; 20 mM EDTA and 1 M NaCl). The mixture was incubated for 1 h at 30 °C and then centrifuged for 5 min at 5000 g. The pellet was washed three times with high-salt buffer (20 mM TRIS–HCl, pH = 8; 20 mM EDTA and 1 M NaCl) to remove non-specifically bound proteins. The resin mixture was then washed with equilibration buffer and finally resuspended in 100 µl Laemmli buffer (Bio-Rad), boiled 5 min at 100 °C, centrifuged for 5 min at 750g and then subjected to SDS-PAGE as previously described by Lee et al. (2012)^15^.

### Computational assessment of the SlHBP2 heme-binding region

The 3D model of SIHBP2 was obtained from AlphaFold, available at: https://alphafold.ebi.ac.uk/entry/A0A3Q7HE03^19^. Structure quality was assessed based on the pLDDT score of AlphaFold where regions having low to very low model confidence (pLDDT < 70) were colored grey and regions having high to very high model confidence (pLDDT > 70) were colored black on the full amino acid sequence. pLDDT, which is scaled from 0 to 100, is an estimate of the confidence of each amino acid corresponding to the model’s predicted score on the lDDT-Cα metric^19^. Only regions with high to very high confidence score are deemed suitable for characterization of ligand or cofactor binding sites, through the subsequent docking simulations. Structure assessment includes the presence of distinct cavity or pocket that can spatially accommodate the heme moiety, and the presence of suitable amino acids at the heme environment such as histidine or tryptophan which are known to participate in iron binding or heme stabilization in hemoproteins such as myoglobins. Molecular docking with SlHBP2 as the receptor and heme as the ligand, were conducted using AutoDock Vina (version 1.1.2)^20^ and structural visualization and image preparation were performed using UCSF Chimera^22^.

### Microbial strains growth conditions

For routine analysis, *Pst* conserved in glycerol stocks at − 80 °C, was grown in KB (King's B) agar media containing 50 μg/mL rifampicin (Sigma-Aldrich) for 24 h, at 28 °C. Additionally, *Xv* and *Cmm* were cultured on NA plates and incubated 24 overnight at 28 °C. *B. cinerea* CECT2100 was purchased from the Spanish collection of type cultures (University of Valencia, 46,100 Burjassot, Spain) was cultured on potato dextrose agar (PDA) (Scharlab) at 24 °C.

### Antimicrobial activity of SlHBP2

#### Activity against *Pst*

Prior to each experiment, *Pst* was grown as described above, and a suspension of 10^8^ Cfu/mL was prepared in MgSO_4_ 10 mM. Experiments were performed in M9 minimal medium prepared as described in Cold Spring Harbor Protocols^59^. M9 minimal medium was supplemented with the purified recombinant protein at 75 µg/mL or with PBS (Thermo Scientific). The pH of the medium was adjusted to 5.8 to mimic the pH of the apoplast, before adding the bacteria. The growth assay was carried out in a Multiskan FC Microplate Photometer (Thermo Scientific) in a total volume of 200 μL in microtiter wells using a final bacterial concentration of 10^6^ Cfu/mL. Bacteria was incubated at 28 °C with continuous agitation and monitored by measuring optical density every 10 min with periodic shaking for 72 h. The same conditions were used to check the antimicrobial activity of the mutated SlHBP2.

#### Activity against *Xv* and *Cmm*

Activity against *Xv* and *Cmm* was tested in a similar way with some minor changes. In brief, experiments were performed in Luria Bertani Broth (LB) (Scharlab) in a total volume of 200 μL in microtiter wells using a final bacterial concentration of 10^4^ Cfu/mL for *Xv* and of 10^5^ Cfu/mL for *Cmm*. LB was supplemented with the purified recombinant protein at 75 μg/mL or with PBS.

#### Activity against *B.cinerea*

*B. cinerea* was cultivated on PDA plates, and spores were collected from 15-day-old plates and diluted in half-strength Potato Dextrose Broth (PDB). The antifungal assay was conducted in 96-well microtiter plates containing PDB. SlHBP2 was used as the antimicrobial agent at the final concentration of 75 µg/mL, while PBS was used as the control. Furthermore, the final fungal suspension was adjusted to a concentration of 1 × 10^4^ spores/mL. The microplate was incubated for 5 days at 25 °C with continuous agitation. Optical density measurements were taken every 30 min throughout the 5-day period with periodic shaking.

### Determination of the minimal inhibitory concentration (MIC) against *Pst*

For *Ps*t, MIC was determinated. The MIC was defined as the lowest concentration that inhibited the visible growth of the bacteria. For this purpose, the bacterium was grown as described above in Antimicrobial activity. Each plate included positive control with M9 with PBS and *Pst*, negative control (M9 with PBS) and SlHBP2 containing wells at the 1.5 µg/mL, 3 µg/mL, 6 µg/mL, 12.5 µg/mL and 25 µg/mL and *Pst*.

### Live/dead viability assay

The cell lysis of *Pst* treated with 1.5, 3, 6, 12.5, and 25 µg/mL of SlHBP2 or with PBS as a control, was assessed by flow cytometry, using LIVE/DEAD™ BacLight™ Bacterial Viability Kit (Molecular Probes, Invitrogen, and Paisley, UK). Then, for live and dead cell quantification, 50 µL of bacterial suspension were mixed with 25 µL of each of the two components of the LIVE/ DEAD BacLight kit and incubated in the dark for 20 min. This bacterial viability kit was used in flow cytometry (BD AccuriTM C6) using the CellQuest software, which was also used to determine the percentage of live/dead cells. While SYTO9 (Live) was excited at 480 nm and fluorescence was analyzed at 500 nm, Propidium Iodide (Dead) was excited at 490 nm and fluorescence analyzed at 635 nm.

### Confocal microscopy

For confocal microscopy, assays were performed as described above in Antimicrobial activity of SlHBP2**.** The microtiter plates containing *Pst* cells treated with SlHBP2 or with PBS were incubated for 16 h at 28 °C using the Multiskan microplate reader. Then the cells were centrifuged and treated with 0.1% Triton X-100 in PBS. The solution was further incubated for 5 min at room temperature (a non-Triton-treated control experiment was also carried out by incubation with PBS alone). Afterwards, the cells were washed twice with PBS, resuspended in 10 µL of PBS, and incubated at room temperature for 2 h with His-Tag antibody conjugated to Alexa Fluor® 647 (Santa Cruz Biotechnology). Then, 1 µL of Hoechst 33,342 staining solution prepared at 1 µg/mL, was added to the mixture, and incubated for 15 min. The cells were washed again in PBS, resuspended in PBS and used to prepare slides for visualization by inverted Confocal Microscope Leica TCS SP8 (Leica Microsystems, Wetzlar, Germany) employing the argon laser's 410–469 nm ray line for its excitation.

### Sample preparation for Raman spectra measurements

*Pst* cells were collected 16 h after incubation with and without SlHBP2, in a 96-well plate and an aliquot of 5 μL of the mixture was dropped onto a glass slide for the Raman spectra measurements. The interactions between *Pst* and SlHBP2 were studied on a slide which was placed in a Raman spectrometer equipped with an Apyron Confocal Microscope (WITec GmbH) with 100x/0.9 NA objective lens (Zeiss) and a 600 lines/mm grid. For excitation, a DPSS laser (532 nm) with a spectral resolution of 8.1 cm^−1^ was used. The laser power at the sample surface was 40 mW. Samples were loaded onto an XYZ moving stage with a minimum step size of 100 µm capacity. In each sample, an area of 22 × 12 µm was measured continuously (24 × 18 points, 3 s per point). All experiments are performed three times.

### Scanning electron microscopy (SEM)

Bacterial samples from the different treatments were harvested by centrifugation and immersed immediately into fixative Karnosky solution containing 0.5% (vol/vol) glutaraldehyde and 2.5% (vol/vol) paraformaldehyde in 0.1 M phosphate buffer (pH 7.4) at room temperature for 1 h and then stored at 4 °C. Samples were postfixed in 2% osmium tetroxide for 2 h and then washed three times in distilled water. After washing, cells were dehydrated in a graded ethanol series (30, 50, 70, 96 and 100%) and then critical-point-dried in a Tousimis CO_2_ chamber model Autosamdry. The filters were then coated with AuPd in a Quorum model SC7640 sputtering apparatus for examination with a Hitachi S4800 SEM with 1.4 nm resolution at 1 kV.

### Bacterial RNA extraction and cDNA synthesis

To determine gene transcription which is involved in the pathogenesis and survival of the *Pst* such as *psyl* gene responsible of the synthesis of the QS signal molecule, *fliC* genes which encodes flagellin, *cfa*, *cmaB* and *cfl* genes involved in synthesis of coronatine, *hrpL* and *hrpA* genes which are involved in the synthesis of the T3SS and pillus formation as well as the gene that encodes the synthesis of the AvrPtoB effector were analyzed. *Pst* were grown overnight in King’s B medium and resuspended in MgSO_4_ (10 mM), adjusting the bacterial cells to an initial concentration of 10^9^ Cfu/mL. Bacteria were grown under the same conditions as described above. Bacterial suspensions were placed in Eppendorf tubes and stabilized with RNA protect bacteria reagent (Qiagen) before centrifugation. Cells pellets were immediately frozen in liquid nitrogen and kept at − 80 °C prior to RNA extraction. Total RNA was extracted by using RNeasy Mini Kit (QIAGEN) and treated with RNase free DNase (Promega) to remove DNA. RNA quantification, and reverse transcription were performed as described in Scalschi et al.^50^. Quantitative polymerase chain reaction (PCR) was conducted using the StepOneTM Real-Time PCR System (Thermo Fisher Scientific, Waltham, MA, USA). Primers used for qPCR are listed in Scalschi et al.^17^. *recA* was used as an internal reference gene to normalize the gene expression in *Pst*.

### Biofilm formation

*Pst* biofilm formation was formed on pre-sterilized 96-well polystyrene microtitre plates. After 72 h of incubation in M9 minimal medium, with and without SlHBP2 (75 µg/mL), the plates were washed three times with distilled water and dried to ensure no residual water remained. Subsequently, the wells were stained with 200 μl of a 1% crystal violet solution. Following an 1 h incubation period, excess crystal violet was removed by gently washing the plates three additional times. The plates were then left to air-dry, allowing the biofilm to adhere firmly to the wells. Once dried, the cell-bound crystal violet was dissolved in 96% ethanol, and biofilm growth was assessed by measuring the OD550nm using a Multiskan microplate reader.

### Data analysis

Statistical analysis was conducted using Minitab 20 software, and the data were expressed as mean ± SE for three replicates per treatment. Normality testing was performed, and significance was determined using Student's t-test or one-way ANOVA, followed by Fisher's LSD at the 95% confidence level (*P* < 0.05) for parametric data and the Kruskal–Wallis test for non-parametric data.

### Supplementary Information

Supplementary Information.

## Data Availability

Data from the study are available from the corresponding author on reasonable request.
