# Supplementary material for: Solanum lycopersicum heme-binding protein 2 as a potent antimicrobial weapon against plant pathogens
Source: Sci Rep. 2023 Nov 20;13:20336. doi: 10.1038/s41598-023-47236-z (PMC10663603; doi:10.1038/s41598-023-47236-z)
Supplement: Supplementary file 1 — Supplementary Information. [file 41598_2023_47236_MOESM1_ESM.docx]

**Supplementary information**

***Solanum lycopersicum* Heme-Binding Protein 2 as a Potent Antimicrobial Weapon Against Plant Pathogens**

**Atefeh Farvardin^1^, Eugenio Llorens^1^, Luisa Liu-Xu^1^, Lorena Sánchez­-Giménez^1^, Aloysius Wong^2^, Elena Biosca^3^, José Pedra^4^, Eva Falomir^5^, Gemma Camañes^1^, Loredana Scalschi^1^*, Begonya Vicedo^1^.**

**^1^**Biochemistry and Biotechnology Group, Department of Biology, Biochemistry and Natural Sciences, Jaume I University, Castellón de la Plana, Spain

^2^College of Science, Mathematics and Technology, Wenzhou-Kean University, 88 Daxue Road, Ouhai, Wenzhou 325060, Zhejiang, China

^3^Department of Microbiology and Ecology, University of Valencia, Burjassot, Spain

^4^Central Service of Scientific Instrumentation. Jaume I University, Castellón de la Plana, Spain

^5^Department of inorganic and organic chemistry, Jaume I University, Castellón de la Plana, Spain


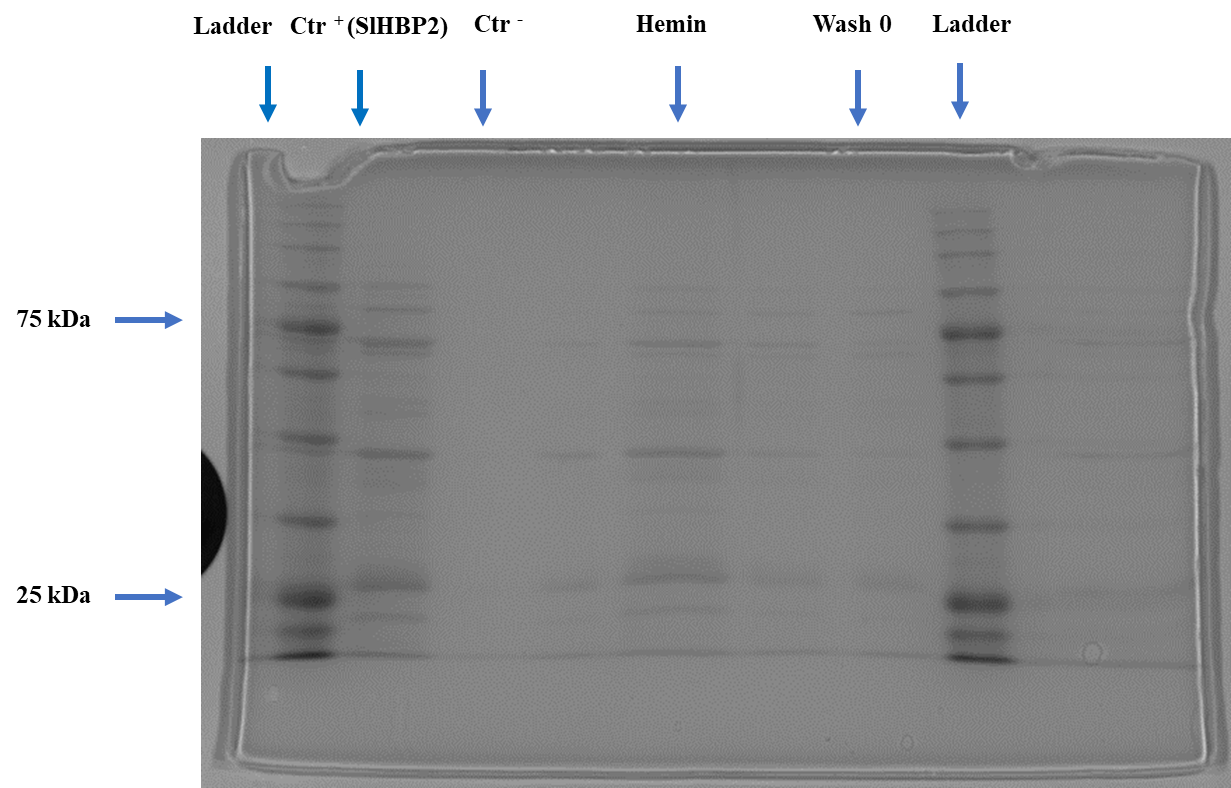


**Supplementary Figure S1.** Heme-binding activity of recombinant SlHBP2.


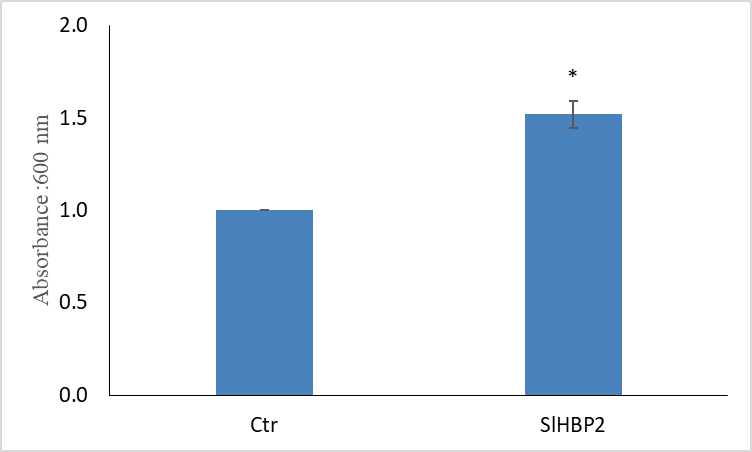


**Supplementary Figure S2**. The biofilm activity of *Pst* in the presence of SlHBP2 was evaluated by staining the biofilms with 1% crystal violet and quantifying them through destaining with 96% ethanol. The absorbance of the destained solution was measured at 550 nm. To assess the statistical significance between groups, the Kruskal-Wallis test was used. The error bar on the graph represents the standard error of the mean, calculated from a minimum of three repetitions.

| gene | Gene Forward primer (5’-3’) | Reverse primer (5’-3’) | Tm(°C) | Product size  (bp) |
| --- | --- | --- | --- | --- |
| SlHBP2 | CTCGAG ACA AAA TTA GAT TCA TAT CCT CCA ACT | GGATCCTCAAATGTCAATTGCAGTGTCC | 62.4/61 | 609 |
| W57 | CTCGAG ACA AAA TTA GAT TCA TAT CCT CCA ACT | GGTTCAGTAGACATCAACATAGGGG | 62.4/56.8 | 125 |
| W211 | CCCCTATGTGGATGTCTACTGAACC | GGATCCTCAAATGTCAATTGCAGTGTCCATAACAAAAGTAAACAACATC | 58.4/64 | 512 |

### Supplementary Table S1. Sequence of primers used for protein production and site-directed PCR mutagenesis.
